# MiRNA-17 encoded by the miR-17-92 cluster increases the potential for steatosis in hepatoma cells by targeting CYP7A1

**DOI:** 10.1186/s11658-018-0083-3

**Published:** 2018-04-18

**Authors:** Ruijie Gong, Xiaofei Lv, Fengqiong Liu

**Affiliations:** 1 Fujian Provincial Key Laboratory of Environment Factors and Cancer, School of Public Health, Fujian Medical University, Fuzhou, Fujian 350008 People’s Republic of China; 2 Department of Epidemiology and Health Statistics, School of Public Health, Fujian Medical University, Fujian, China; 3 Department of Internal Medicine, Guangdong Women and Children’s Hospital, Guangzhou Medical University, 521 Xingnan Road, Guangzhou, China

**Keywords:** MiRNA-17, Steatosis, Fatty liver, CYP7A1

## Abstract

**Background:**

The miRNA cluster miR-17-92 is known to act as an oncogene in various cancers. Members of this cluster were also found to be involved in some other pathological process, such as steatosis, which is a pivotal event in the initiation and progression of nonalcoholic fatty liver disease (NAFLD). This study aimed to explore whether miR-17, one of the most functional miRNAs in the miR-17-92 family, participates in the process of steatosis in hepatoma cells.

**Methods:**

We developed both a miR-17-expressing transgenic mouse model and a miR-17-expressing HepG2 cell model, the latter was established via stable transfection. Real-time PCR and western blot were applied to measure the expression levels of miR-17 and the potential target gene CYP7A1. The luciferase assay was used to confirm direct binding of miR-17 and CYP7A1. The oleic acid induction assay and Oil-Red-O staining were performed to support the determination of steatotic changes in HepG2 cell.

**Results:**

Extensive steatotic changes were observed in the livers of transgenic mice. Fewer were seen in the wild-type animals. CYP7A1 was confirmed as a target gene of miR-17, and the expression of CYP7A1 was found to be negatively regulated in both the transgenic mice liver cells and the miR-17-expressing HepG2 cells. CYP7A1 was found to participate in miR-17-induced steatosis, as its repressed expression in miR-17 HepG2 cells exacerbated steatotic change. Re-introduction of CYP7A1 into miR-17 HepG2 cell partially alleviated steatosis.

**Conclusions:**

miR-17 is a novel regulator of CYP7A1 signaling in hepatic lipid metabolism, suggesting a potential therapeutic approach for fatty liver.

**Electronic supplementary material:**

The online version of this article (10.1186/s11658-018-0083-3) contains supplementary material, which is available to authorized users.

## Background

Nonalcoholic fatty liver disease (NAFLD) represents a spectrum of metabolic syndrome-associated liver pathologies progressing from simple steatosis through nonalcoholic steatohepatitis (NASH) and fibrosis to cirrhosis and hepatocellular carcinoma (HCC) [1]. It is the most common causes of chronic liver disease worldwide and it is closely associated with metabolic syndrome [2]. Although its risk factors have been clearly defined, the underlying mechanisms of its progression remain poorly understood [3]. The general pathogenesis involved includes insulin resistance, steatosis, oxidative stress, inflammation, hepatocyte injury and apoptosis, fibrosis, and carcinogenesis [4, 5]. Steatosis is the initial stage — and a vital part — of NAFLD development [6].

MicroRNAs (miRNAs) are small noncoding RNAs that negatively regulate target gene expression through base pairing with 3’ UTRs inducing mRNA cleavage or translational repression. The specific contribution of selected microRNAs in hepatic disease development and progression has been described [7]. miR-122 is one of the most abundant miRNAs in the liver. Its inhibition in normal mice resulted in reduced plasma cholesterol levels, increased hepatic fatty acid oxidation, and decreased hepatic fatty acid and cholesterol synthesis rates [8]. Aberrant upregulation of miR-21 expression due to excessive circulating levels of fatty acids exemplifies a recently discovered regulatory mechanism by which fatty acids affect PTEN expression and trigger liver disorders [9]. miR-467b expression is significantly downregulated in liver tissues of mice fed a high-fat diet and in steatosis-induced hepatocytes. The downregulation of miR-467b resulted in the upregulation of hepatic lipoprotein lipase (LPL) [10].

Unique miRNA profiles were identified as responsible for the transition from hepatic steatosis to steatohepatitis, and they can be used diagnostically differentiate steatohepatitis from steatosis. Through KEGG (Kyoto Encyclopedia of Genes and Genomes) analysis, 46 and 41 enriched pathways were collected for the target genes of up- and downregulated miRNAs. Those pathways included apoptosis, fatty acid metabolism and so on [11], suggesting that miRNA might also regulate steatosis formation in NAFLD through direct targeting of downstream molecules.

miR-17-92, one of the best-characterized miRNA clusters, is well known for its important role as an oncogene in various cancers as well as in mammalian development and cell differentiation [12–14]. miR-17 upregulates the migration and proliferation of HCC cells by activating the p38 mitogen-activated protein kinase (MAPK) pathway It also increases the phosphorylation of heat shock protein 27 (HSP27) [15]. miR-17 was significantly upregulated in hepatocellular carcinoma samples compared with its level in paracarcinomatous liver tissues. It was shown to be an independent risk factor for overall survival and disease-free survival [16]. miR-17 also suppresses cell proliferation and invasion by targeting ETV1 in breast cancer [17]. It was also reported to target the p300/CBP-associated factor and modulate androgen receptor transcriptional activity in prostate cancer cells [18]. Besides its critical role in the progress of hepatic carcinoma, miR-17 is also involved in some other pathophysiologic processes. It regulates 3 T3-L1 cell adipogenic differentiation by inhibiting and stimulating the Wnt signaling pathway effector Tcf7l2 [19]. Circulating miR-17 was found to be higher in patients with coronary artery disease and to be associated with the blood lipid levels in those patients [20].

In a previous study, an miRNA-17 transgenic mouse model was established [21].We observed that expression of miR-17 retards early-stage tissue growth in these transgenic mice. To examine how the expression of miR-17 can affect the metabolism of mouse tissue, organs from transgenic mice were examined and extensive steatotic changes were found in the liver. We hypothesize that miR-17 may play an important role in energy homeostasis and lipid metabolism in hepatocytes and thus lead to steatosis and fatty liver.

In this study, we investigated the potential mechanism by which miR-17 may mediate hepatosteatosis. The aim is to deepen the understanding of the molecular basis of these diseases and to identify new therapeutic targets.

## Methods

### Generation and genotyping of miR-17 transgenic mice

Transgenic mice expressing miR-17 were generated as described previously [21]. A fragment of cDNA harboring four copies of pre-miR-17 was generated to enhance miR-17 expression. The resulting miR-17 construct was digested with ApaI and StuI to release the miR-17 transgene followed by microinjection into the oviducts of pseudo-pregnant recipient females using a standard protocol approved by the Animal Care and Use Committee of Fujian Medical University. Transgenic founder lines were maintained by backcrossing with C57BL/6F1 mice.

### Construct generation

A cDNA sequence containing two pre-miR-17 units was inserted into the mammalian expression vector pEGFP-N1 in the restriction enzyme sites BglII and HindIII. This plasmid was expected to simultaneously express miR-17 and green fluorescent protein (GFP). A luciferase reporter vector (psiCHECK-2; Promega) was used to generate the luciferase constructs. The 3′ untranslated region (3’UTR) of CYP7A1 was synthesized and then digested with XhoI and NotI and the fragments were inserted into a XhoI- and NotI-opened psiCHECK-2 luciferase vector to obtain the luciferase construct psiCHECK-CYP7A1. To serve as a negative control, we synthesized the 3’UTR of CYP7A1 with the miR-17 binding sites replaced by a random nucleotide sequence. This was cloned to the psiCHECK-2 luciferase vector to obtain the mutant luciferase construct psiCHECK-CYP7A1 mut. The coding sequence of CYP7A1 (2875 bp) was synthesized and digested with EcoRI and XhoI, then the fragments were inserted into an EcoRI- and XhoI-opened pcDNA3.1 vector to obtain the CYP7A1-expressing construct.

### Oil-red-O staining

Frozen liver sections were fixed in 10% formalin and briefly washed with water for 10 min. The sections were rinsed with 60% isopropanol and stained with freshly prepared Oil-Red-O working solution for 15 min. Then, the sections were rinsed with 60% isopropanol, lightly stained with aluminium haematoxylin, and mounted in glycerine jelly.

### Liver triglyceride content measurement

Liver samples were weighed (100–300 mg) and 300 μl of ethanolic KOH (2 parts EtOH mixed with 1 part 30% KOH) were added to each sample, followed by incubation at 55 °C overnight. Diluted EtOH (1:1 H_2_O:EtOH) was added to each tube to bring the volume to 1 ml. The tubes were centrifuged for 5 min and the supernatant was transferred into new tubes. The diluted EtOH was added again into each tube to bring the volume to 1.2 ml. After vortexing, 200 μl of the mixture was transferred into new Eppendorf tubes in triplicates, followed by the addition of 215 μl 1 M MgCl_2_ and incubation on ice for 10 min. The tubes were centrifuged for 5 min and the supernatant was transferred into new tubes. Triglyceride reagent (Sigma, catalog number F6428) was reconstituted according to manufacturer’s instructions for the determination of glycerol content. Then, 1 ml reconstituted reagent was pipetted into each cuvette. Liver lysates, standards and control blanks were added into separate cuvettes, and incubated at 37 °C for 15 min. Absorbance values were measured at 540 nm.

### Induction of steatosis in HepG2 cell by oleic acid treatment

HepG2 cells were cultured in 96-well tissue culture plates to sub-confluence. The medium was changed for serum-free DMEM, followed by overnight treatment of the cells with 200 μl oleic acid (OA) solution at concentrations from 0 to 2 mM.

### Oil-red-O-based steatosis quantification

Steatosis was induced in HepG2 cells using OA. The cells were then stained with Oil-Red-O as described above. After washing and completely drying, 100 μl of 100% isopropanol was added to each well, followed by incubation at room temperature for 10 min to release the Oil-Red-O from the steatosis staining. The extraction solution was transferred to another 96-well plate which was subjected to OD measurement at a wavelength of 490 nm using a microplate reader (Bio-Tek Instrument Inc.).

### Western blot

Liver tissues and harvested cells were lysed in RIPA buffer with a protease inhibitors cocktail (Roche). Total protein (30 mg) was separated via 12% SDS-polyacrylamide gel electrophoresis, transferred to a polyvinylidene fluoride membrane, and hybridized with anti-CYP7A1 and anti-β-actin (Santa Cruz Biotechnology) overnight at 4 °C. After incubation with horseradish peroxidase-conjugated secondary antibody (Santa Cruz Biotechnology) for 2 h at room temperature, immunoreactive proteins were visualized with the West-one Western Blot Detection System.

### Luciferase assay

HepG2 cells were cultured in a 24-well tissue culture plate at a density of 3 × 10^4^ cells per well. The cultures were maintained at 37 °C for 24 h, and then the luciferase reporter constructs psiCHECK-CYP7A1 or psiCHECK-CYP7A1 mut were co-transfected with miR-17 mimics, inhibitors or mock miRNAs (Shenggong) using Lipofectamine 2000. The cells were then collected and lysed with a luciferase-specific lysis buffer from a Luciferase Assay Kit (Promega). Luciferase activity was measured using the Dual-Luciferase Reporter Assay System (Promega) and normalized to the activity of renilla luciferase.

### Real-time PCR

Quantification of CYP7A1 mRNA transcripts (forward primer: ATCTGGAGAAGGCCAAGACA, reverse primer: TTTCATTGCTTCTGGGTTCC) was performed via SYBR Green quantitative real-time PCR using the ABI Prism 7500 sequence detection system (Applied Biosystems) with normalization to the expression of GAPDH. miR-17 (primer sequence: CAAAGTGCTTACAGTGCAG) was detected with specific stem-loop primers using a miScript Reverse Transcription Kit (Qiagen) and miScriptSYBR Green PCR Kit (Qiagen).

### Statistical analysis

For the statistical analysis, SPSS19.0 software was used. One-way analysis of variance (ANOVA) or Student’s *t* test were used to compare quantitative data among groups. Bonferroni’s post hoc test was used if ANOVA indicated significance (*p* < 0.05). All data are shown as means ± S.D.

## Results

### Development of fatty liver in miR-17 transgenic mice

Organs from both wild-type and transgenic mice (20 for each group) were examined. Expression of miR-17 was higher in transgenic mice than in wild-type mice (Additional file 1: Figure S1) and stable in the transgenic mouse model (Additional file 2: Figure S4).

**Fig. 1:**
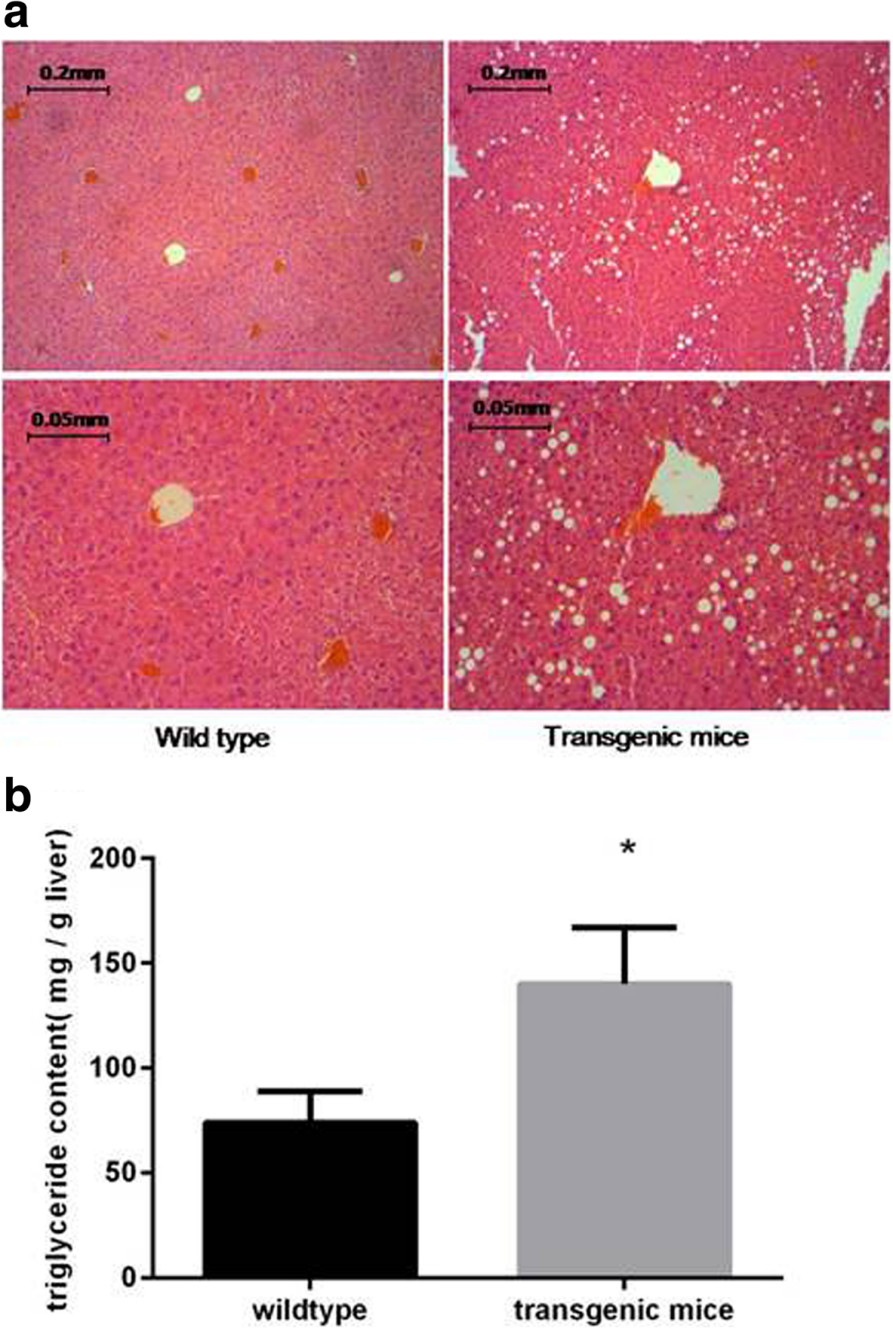
miR-17 transgenic mice exhibit extensive steatotic changes in their livers. **a** Hematoxylin-eosin staining shows excessive lipid accumulation in the liver of transgenic mice. **b** Quantitative analysis of liver triglyceride shows that the transgenic mouse liver exhibits significantly higher levels of triglyceride than the wild-type mouse liver (*n* = 10 for each group)

Histological analyses showed extensive steatotic changes in the livers of transgenic mice but fewer in the wild-type mice (representative results in Fig. 1a). Triglyceride content was analyzed in liver tissue from 10 randomly selected transgenic mice and 10 wild-type mice. The transgenic mouse livers had significantly higher levels of triglyceride than the wild-type mouse livers (Fig. 1b).

### MiR-17 regulates the steatosis level in HepG2 cell

**Fig. 2:**
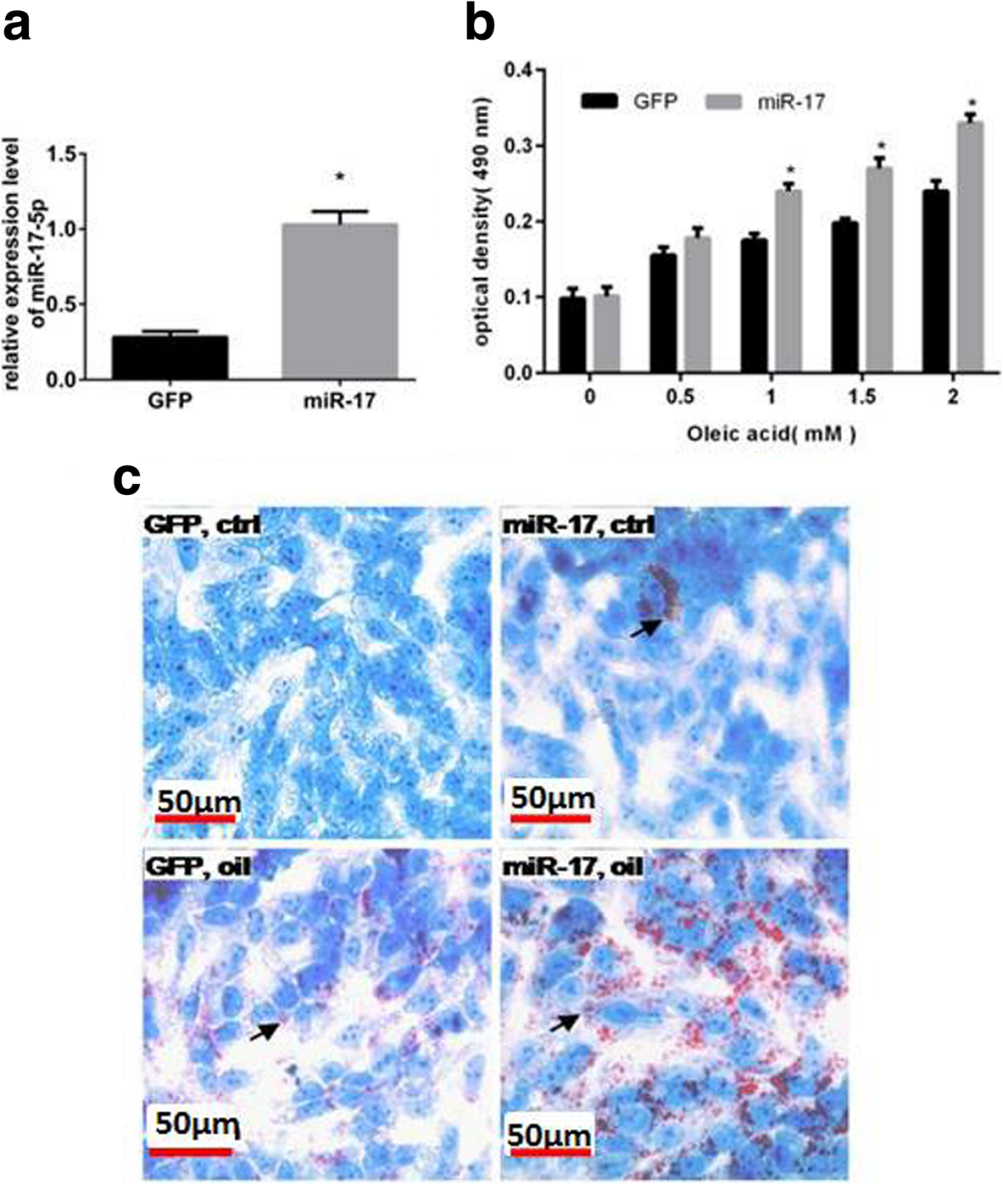
miR-17 induces steatosis in HepG2 cell. **a** miR-17 overexpression in HepG2 cells stably transfected with miR-17. **b** Transfection of miR-17 enhanced oleic acid (OA) uptake. **c** Representative pictures of Oil-Red-O staining of HepG2 cells. “GFP” (green fluorescent protein) represent native control plasmid which didn’t express miR-17. “ctrl” represent cell groups without oleic acid treatment, while “oil” represent cell groups with oleic acid treatment

To further analyze the role of miR-17 in inducing steatosis in liver, HepG2 cells were stably transfected with an miR-17-expressing construct or GFP construct. The miR-17 overexpression was confirmed using real time-PCR (Fig. 2a). Then we treated miR-17- or GFP-transfected HepG2 cells with oleic acid followed by Oil-Red-O staining to reveal steatosis. Transfection with miR-17 enhanced oleic acid uptake (Fig. 2b and c).

### CYP7A1 is a direct target of miR-17

**Fig. 3:**
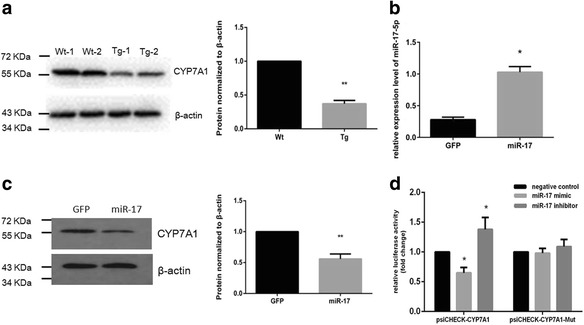
CYP7A1 is a direct target of miR-17. **a** Western blotting showed CYP7A1 expression was repressed in the liver of miR-17 transgenic mice. **b** Expression of CYP7A1 mRNA decreased in miR-17 HepG2 cells. **c** Protein expression of CYP7A1 was decreased in miR-17 HepG2 cells. **d** Luciferase activity changed in human psiCHECK-CYP7A1 and miR-17 co-transfection but not for psiCHECK-CYP7A1 mutant

Bioinformatic analysis indicated that miR-17 potentially targets many mRNAs. We focused on those that were known to be important in cell lipid metabolism and in the development of a fatty liver. Extensive analysis with western blotting revealed that CYP7A1 was of potential interest (Additional file 3: Figure S2). It was found to be repressed in the miR-17 transgenic mice compared with its expression in wild-type mice (Fig. 3a). The CYP7A1 expression level was stable in the transgenic mouse model (Additional file 4: Figure S5).

The targeting between CYP7A1 and miR-17 is conserved between mouse and human models (Additional file 3: Figure S2). We confirmed the effect of miR-17 on CYP7A1 expression in the HepG2 cell line. Using western blot and real time-PCR, the protein and mRNA levels of CYP7A1 were analyzed in HepG2 cells stably transfected with miR-17 or GFP. There was significant decrease in the mRNA (Fig. 3b) and protein expressions (Fig. 3c) in the miR-17 transfected cells.

To further confirm that CYP7A1 was a target of miR-17 in humans, we generated a luciferase construct (psiCHECK-CYP7A1) harboring the binding sites of miR-17 and constructs in which miR-17 target sites were mutated (psiCHECK-CYP7A1 mut HepG2 cells were co-transfected with either CYP7A1 3’UTR luciferase constructs (psiCHECK-CYP7A1) or mutant constructs (psiCHECK-CYP7A1 mut), along with miR-17 mimics or inhibitors. Significant repression of luciferase activity was detected in psiCHECK-CYP7A1-transfected cells. Mutation of the binding sites reversed this repressive effect of miR-17 (Fig. 3d). Similarly, a luciferase construct harboring the binding sites of mouse miR-17 was generated and the luciferase assay was performed to verify that the binding between miR-17 and CYP7A1 is conserved (Additional file 5: Figure S3).

### Confirmation that CYP7A1 can mediate miR-17-induced steatosis

**Fig. 4:**
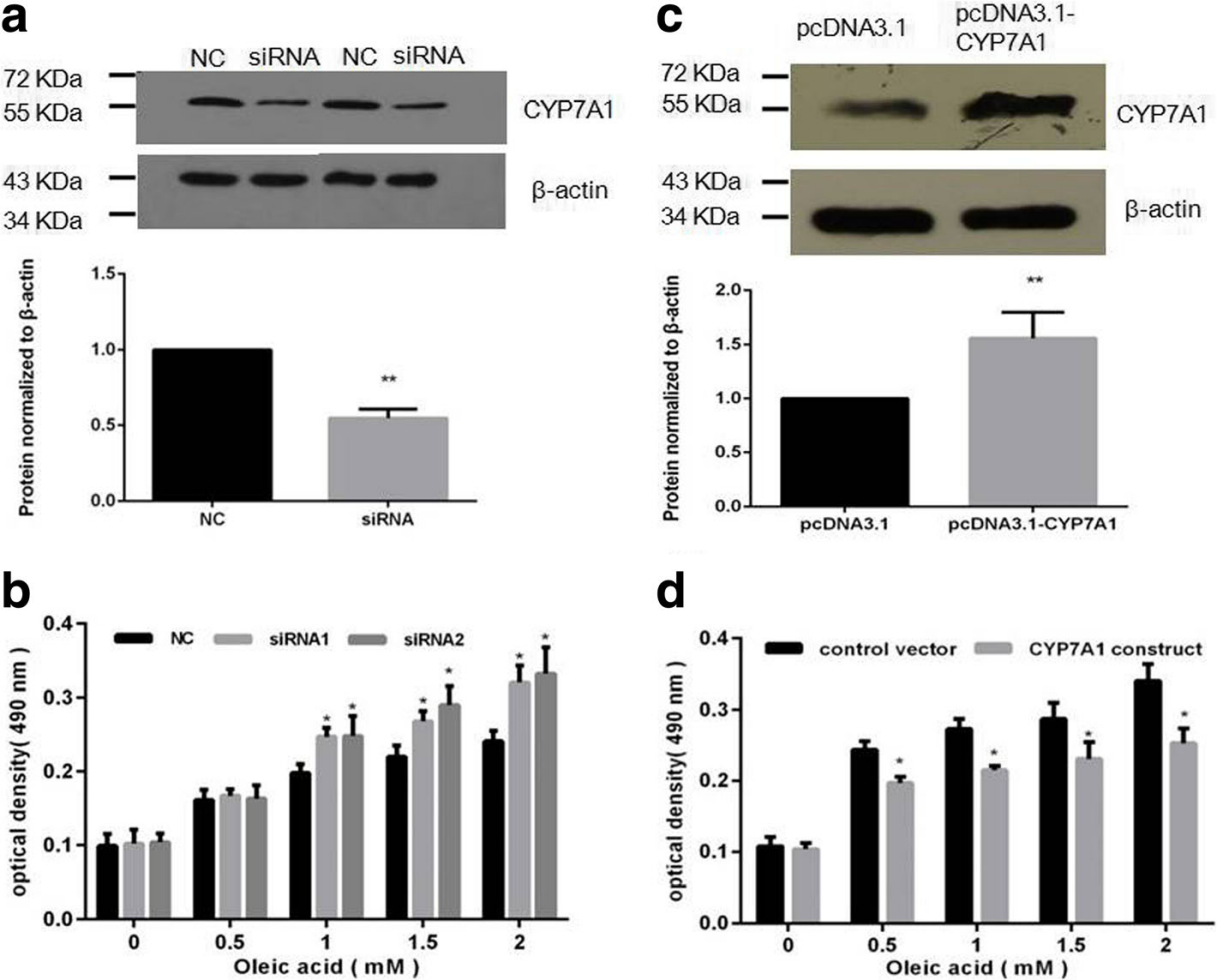
miR-17-induced steatosis depends on the expression of CYP7A1. **a** Downregulation of CYP7A1 by siRNA transfection in HepG2 cells. **b** CYP7A1 siRNA transfection promotes steatosis in HepG2 cells. **c** Upregulation of CYP7A1 by CYP7A1 expression construct transfection in miR-17 HepG2 cells. **d** Re-introduction of CYP7A1 into miR-17 HepG2 cells could partially alleviate steatosis

To corroborate that CYP7A1 can contribute to cell steatosis, we generated two small interfering RNA (siRNA) constructs targeting CYP7A1. Downregulation of CYP7A1 was detected using western blot in the siRNA-transfected HepG2 cell (Fig. 4a). The steatosis induction assay showed that transfection with CYP7A1 siRNA can promote HepG2 steatosis compared with the impact on cells transfected with control oligos (Fig. 4b). These results indicate that CYP7A1 is associated with HepG2 cell steatosis.

To further confirm that miR-17 targeting of CYP7A1 can affect cell steatosis, rescue experiments were performed. HepG2 cells stably transfected with miR-17 were transiently transfected with CYP7A1 expression constructs and a control vector for the steatosis assay. Upregulation of protein expression after transfection with the CYP7A1 expression construct was confirmed via western blot (Fig. 4c). When HepG2 cells were transfected with the CYP7A1 expression construct after miR-17 overexpression, a significant decrease in steatosis was observed compared with the control vector (Fig. 4d). This further confirmed that the CYP7A1-mediated pathway is essential for miR-17 to affect HepG2 cell steatosis. Our results above indicate that miR-17 induced the development of fatty liver and these effects could partially be attributed to the mediation of CYP7A1.

## Discussion

The miR-17-92 family is one of the most commonly upregulated in human cancers, including HCC, and the members are considered “oncogenic” miRNAs [22, 23]. Recent studies suggest that different miRNAs in the miR-17-92 cluster may have distinct functions [24]. Inhibition of miR-17 and miR-20a in cells overexpressing the miR-17-92 cluster can induce apoptosis, while inhibition of miR-18a and miR-19a did not have the same effect and inhibition of miR-92-1 resulted in only a modest reduction of cell growth [25].

To fully exploit how single members of the cluster can affect physiologic and pathologic processes, we produced a transgenic mouse model that exhibits stable overexpression of miR-17. We found that the livers of transgenic mice exhibited higher levels of steatosis than those of wild-type mice. Additionally, the transgenic animals exhibited a strong predisposition to the development of liver tumors with spontaneously developed visible neoplastic lesions, which were undetectable in the wild-type mice [21].

Steatosis is the earliest stage of NAFLD, which is frequently observed in the context of metabolic dysfunctions that predispose to HCC in humans. Hepatic steatosis is often self-limited, but it can progress to NASH [26]. Since cell injury may occur when the capacity of hepatocytes to safely store fat is overwhelmed by continued uptake, local synthesis or impaired egress of fatty acids, these fatty acids then become toxic to the cell, causing cell death through the direct effects of lipid mediators on apoptosis [27]. Steatosis could therefore provide the setting for NASH, which can lead to hepatic fibrosis or cirrhosis, and finally may proceed to hepatocellular carcinoma [28].

As observed in the liver of some miR-17 transgenic mice, steatosis was accompanied by HCC [21], indicated that steatosis may be a mechanism in the progression of miR-17-mediated HCC. However, those tumors did not arise on the cirrhotic background that is typical of most human HCCs, suggesting that miR-17 can directly cause hepatocellular carcinoma through pathways that are independent of NAFLD. Similar results were also obtained in a study involving a miR-221-overexpressing transgenic mouse model: it was found that the liver tumor-promoting effect of miR-221 was accompanied by extended steatosis [29] .

At the molecular level, the liver of transgenic mice revealed an overexpression of miR-17 that was accompanied by a strong repression of the lipid metabolism-related protein CYP7A1. In the liver, cholesterol is converted to bile acids via a pathway with abundant physiological reactions. CYP7A1 is a liver-specific rate-limiting enzyme of this pathway [30]. CYP7A1 deficiency caused by a homozygous deletion mutation could reduce the conversion of cholesterol to bile acids, resulting in accumulation of cholesterol in the liver, leading to downregulation of LDL receptors and consequent elevated LDL cholesterol and hypertriglyceridemia [31]. Polymorphisms in the promoter of this gene are associated with defects in bile acid synthesis. They affect lipid responses to the lipid-lowering drug fenofibrate [32]. Mice deficient in CYP7A1 also exhibited changes in cholesterol and bile acid metabolism in the liver [33], while overexpression of CYP7A1 in the liver resulted in improved metabolic homeostasis in CYP7A1 transgenic mice [34, 35].

In our study, forced expression of mature miRNA-17 in HepG2 cells significantly reduced the expression of CYP7A1 through direct binding to the 3’UTR, which is vital for efficient activation of downstream pro-lipolytic gene expression. As firmly established in the literature, disruption of CYP7A1 signaling can promote lipid uptake and/or inhibit lipolysis, as evidenced by increased lipid accumulation.

## Conclusions

This study provides the first evidence that miR-17 has a functional role in liver steatosis. Mechanistically, miR-17 acts as a novel inhibitor of CYP7A1 signaling in the hepatocyte and holds clinical promise as a therapeutic molecule for NAFLD.

## Additional files

Additional file 1: **Figure S1.** Expression of miR-17 increases in transgenic mice compared with wild-type mice. (JPG 599 kb)

Additional file 2: **Figure S4.** miR-17 expression level was stable in miR-17 transgenic mice. (JPG 829 kb)

Additional file 3: **Figure S2.** A – CYP7A1 is predicted to be a target of miR-17 in humans. B – CYP7A1 is predicted to be a target of miR-17 in mice. (JPG 87 kb)

Additional file 4: **Figure S5.** CYP7A1 expression level was stable in miR-17 transgenic mice. (JPG 810 kb)

Additional file 5: **Figure S3.** Luciferase activity changed in mouse psiCHECK-CYP7A1 and miR-17 co-transfection but not for psiCHECK-CYP7A1 mutant. (JPG 120 kb)

## Abbreviations

CYP7A1: Cytochrome P450 family 7 subfamily A member 1
GFP: Green fluorescent protein
HCC: Hepatocellular carcinoma
NAFLD: Nonalcoholic fatty liver disease
NASH: Nonalcoholic steatohepatitis
OA: Oleic acid
