# Supplementary figures and images for: MiRNA-17 encoded by the miR-17-92 cluster increases the potential for steatosis in hepatoma cells by targeting CYP7A1
Source: Cell Mol Biol Lett. 2018 Apr 18;23:16. doi: 10.1186/s11658-018-0083-3 (PMC5907481; doi:10.1186/s11658-018-0083-3)

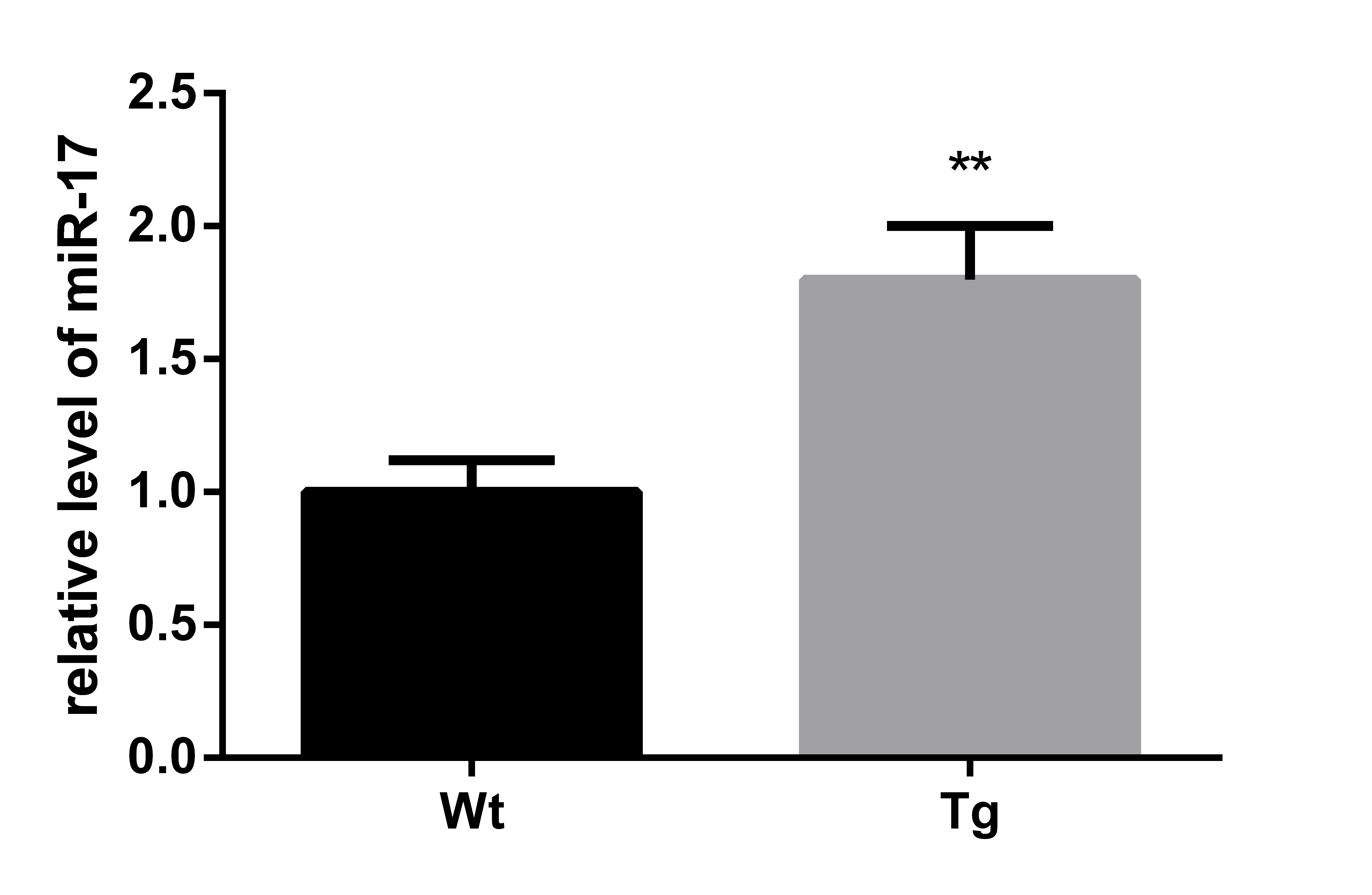

Supplement: Supplementary file 1 — Figure S1. Expression of miR-17 increases in transgenic mice compared with wild-type mice. (JPG 599 kb) [file 11658_2018_83_MOESM1_ESM.jpg]

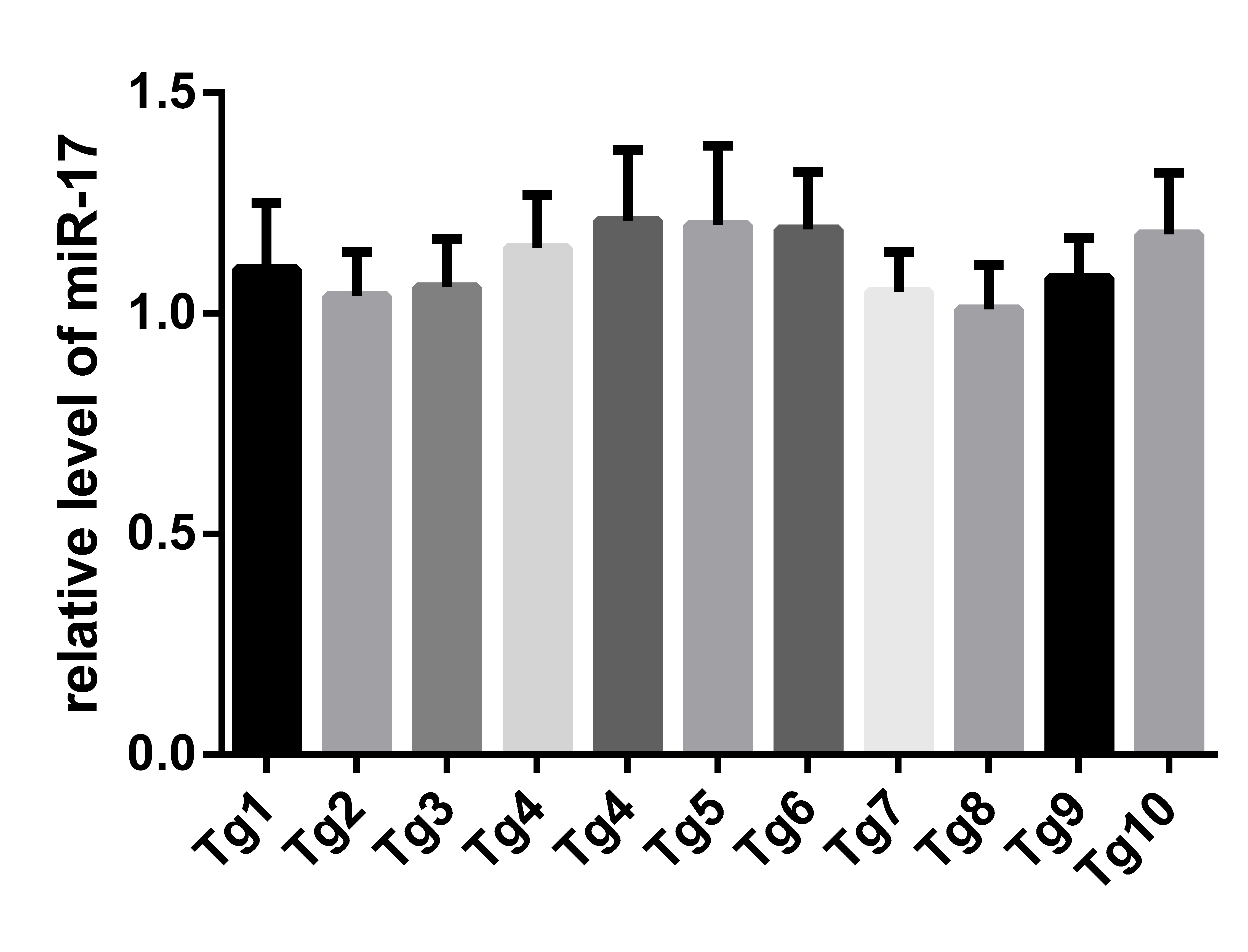

Supplement: Supplementary file 2 — Figure S4. miR-17 expression level was stable in miR-17 transgenic mice. (JPG 829 kb) [file 11658_2018_83_MOESM2_ESM.jpg]

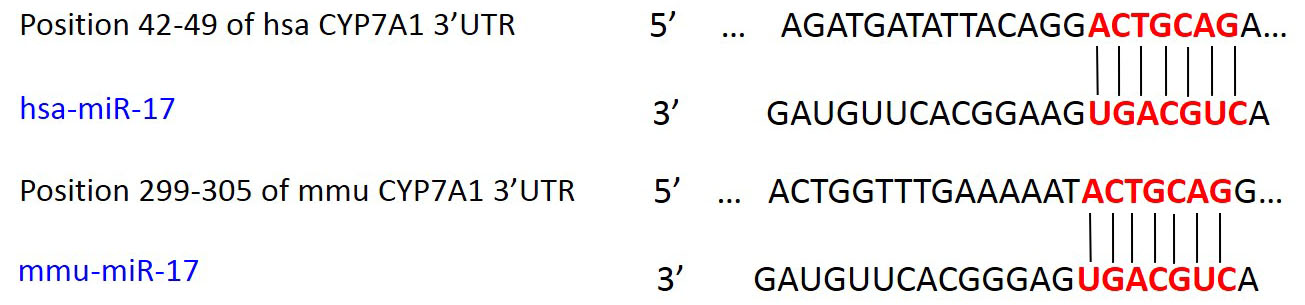

Supplement: Supplementary file 3 — Figure S2. A – CYP7A1 is predicted to be a target of miR-17 in humans. B – CYP7A1 is predicted to be a target of miR-17 in mice. (JPG 87 kb) [file 11658_2018_83_MOESM3_ESM.jpg]

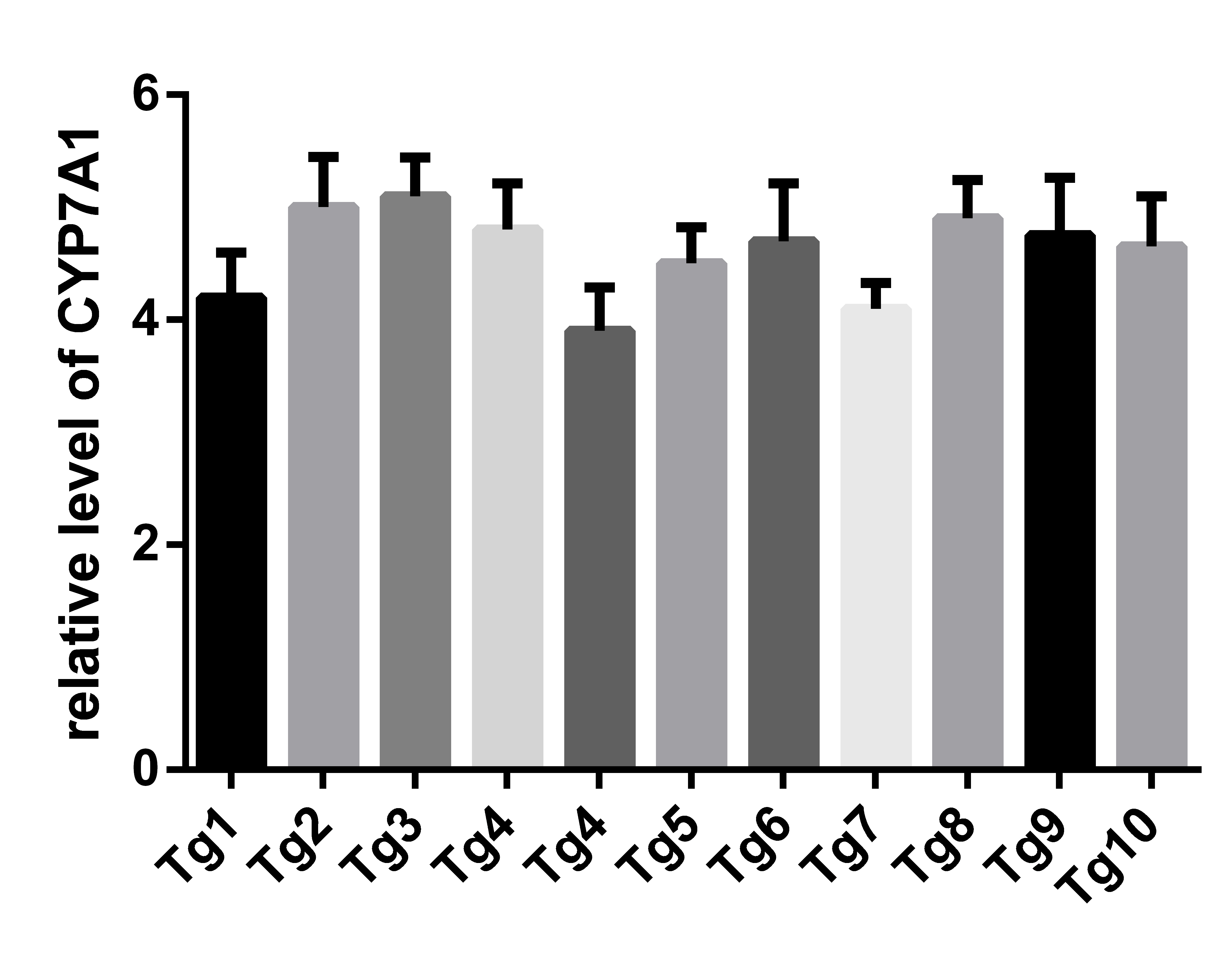

Supplement: Supplementary file 4 — Figure S5. CYP7A1 expression level was stable in miR-17 transgenic mice. (JPG 810 kb) [file 11658_2018_83_MOESM4_ESM.jpg]

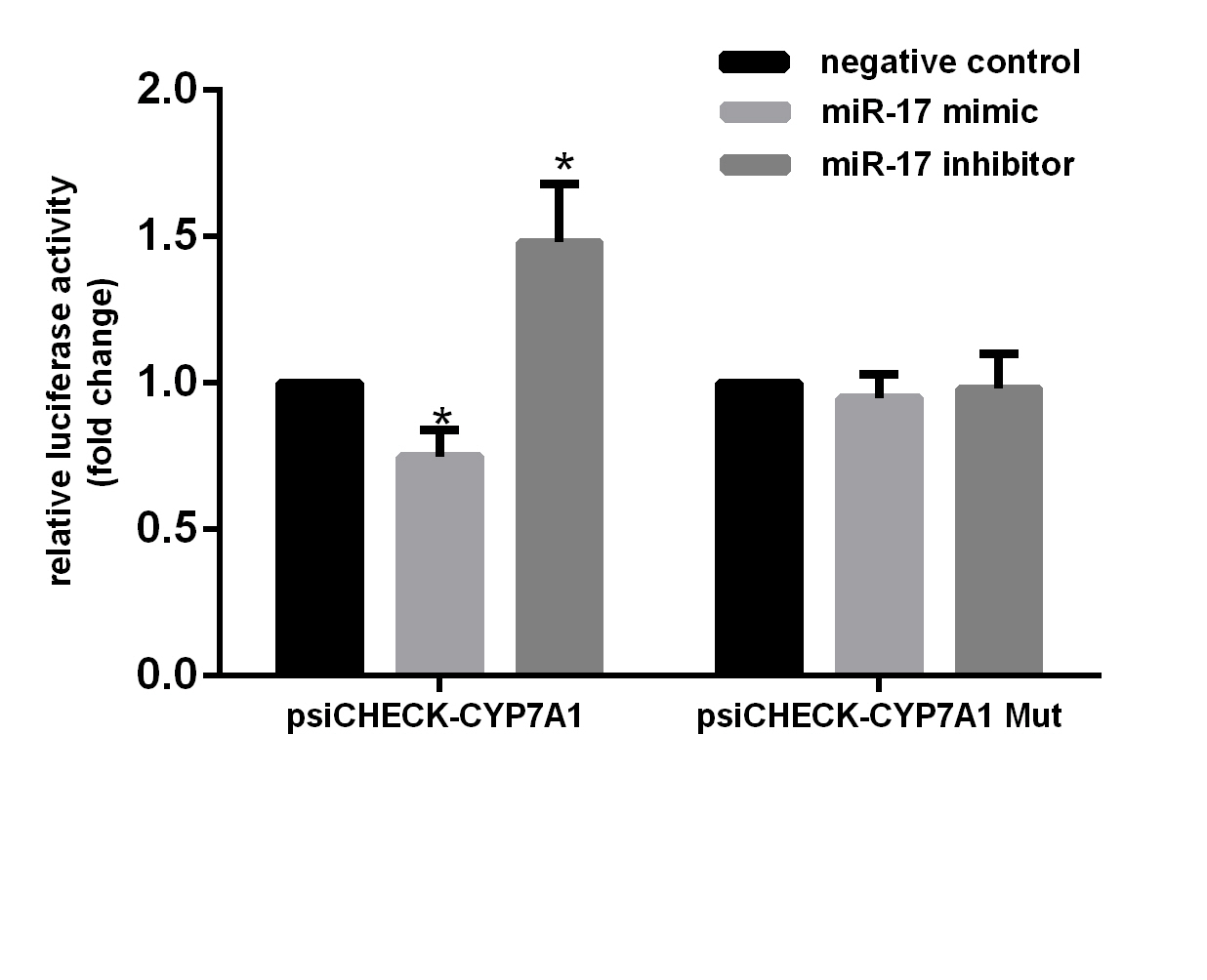

Supplement: Supplementary file 5 — Figure S3. Luciferase activity changed in mouse psiCHECK-CYP7A1 and miR-17 co-transfection but not for psiCHECK-CYP7A1 mutant. (JPG 120 kb) [file 11658_2018_83_MOESM5_ESM.jpg]
